# Factors Associated with Intent to Leave and Burnout among Canadian Nurses Amidst the COVID-19 Pandemic: A Quantitative Analysis of the Survey on Health Care Workers’ Experiences During the Pandemic

**DOI:** 10.1177/08445621251338580

**Published:** 2025-04-29

**Authors:** Kishana Balakrishnar, Bao-Zhu Stephanie Long, Alexia M. Haritos, Edris Formuli, Behdin Nowrouzi-Kia

**Affiliations:** 1 Department of Occupational Science and Occupational Therapy, 12366 Temerty Faculty of Medicine, University of Toronto, Toronto, Ontario, Canada; 2 Krembil Research Institute-University Health Network, Toronto, Ontario, Canada; 3 Centre for Research in Occupational Safety & Health, 7728 Laurentian University, Sudbury, Ontario, Canada

**Keywords:** burnout, COVID-19, intention to leave, nurses, occupational stress

## Abstract

**Background:**

The increased demands and stressors from the COVID-19 pandemic led to widespread burnout and job stress, prompting concerns about retention rates. This study identifies demographic and occupational predictors of Canadian nurses’ intent to leave their jobs due to burnout and job stress during the COVID-19 pandemic.

**Methods:**

Data was utilized from the Survey on Health Care Workers’ Experiences During the Pandemic conducted by Statistics Canada. Multivariate logistic regression models were generated to analyze the associations between demographic and occupational factors and nurses’ intent to leave.

**Results:**

A total of 12,246 eligible participants responded to the survey (54.9% response); however, the analysis was restricted to 1138 nurses after excluding participants of other healthcare occupations. Younger nurses were significantly more likely to consider leaving their jobs [OR = 9.95, 95% CI: (5.92–16.73)], as well as nurses living in Alberta [OR = 3.16, 95% CI: (1.58–6.32)] and British Columbia [OR = 3.16, 95% CI: (1.66–6.03)]. Moreover, nurses with less work experience [OR = 3.91, 95 CI = (2.53–6.05)], work in acute care [(OR = 3.31, 95 CI = (1.69–6.51)], experienced changes in workload [OR = 2.69, 95% CI: (1.58–4.57)], had increased work hours [OR = 1.92, 95% CI: (1.27–2.92)], and lacked emotional support [OR = 3.43, 95 CI = (2.31–5.09)] had greater odds of intending to leave.

**Conclusion:**

The findings underscore the need for strategies to mitigate stress and burnout among nurses, particularly during public health crises. Implementing measures to address these factors could help improve retention rates and ensure a stable nursing workforce during future pandemics.

## Background and purpose

In Canada, nurses form the basis of the health care system and are the largest group of regulated health professionals with over 450,000 members (Health Canada, 2024). Nursing is defined as a healthcare field that “integrates the art and science of caring and focuses on the protection, promotion, and optimization of health and human functioning; prevention of illness and injury; facilitation of healing; and alleviation of suffering through compassionate presence” (American Nurses Association, 2021, p. 1). Nurses play a pivotal role in the health care system as they are at the forefront of detecting health emergencies and providing health services, including treatment, prevention, promotion, and rehabilitation (World Health Organization, 2024). The emergence of the COVID-19 pandemic has further demonstrated the importance of nurses in our healthcare system as they work on the frontlines to prevent, treat, and manage conditions (Smith et al., 2020).

Despite the pandemic highlighting the importance of nurses to the healthcare sector, it posed a significant strain on their mental health, leading to increased job stress and burnout (Crowe et al., 2022; Mary Pappiya et al., 2023; Papazian et al., 2023; Quesada-Puga et al., 2024). The co-occurrence of innumerable deaths and extended work shifts filled with a wide range of uncertainties and demands can contribute to depression and anxiety, which makes occupational stress related to COVID-19 an important marker of mental illness (Esmaeili et al., 2015; Said & El-Shafei, 2021). As nurses exhibit constant, in-depth interactions with patients (Saini et al., 2011; Salehi, 2013), nurses are more susceptible to psychosocial risks (Zakiyah et al., 2022). This is due to the constantly changing traits of SARS-CoV-2 and the potential for infection, whereby the lack of personal supply of safety gear and other medical supplies, insufficient testing, few alternatives for treatment, worries about medication and infection to family members, emotional and ethical engagement all contribute to work-related stress (Giménez-Espert et al., 2020). If the stress levels continue to accumulate (Mo et al., 2020; Zandian et al., 2021), it will hinder the completion of the task, resulting in a lower job contentment of nurses while delivering nursing care (Aziz, 2020). Job discontent and work stress are mutually correlated (Babapour et al., 2022). When employees believe that their compensation is not commensurate with their work, it can lead to discontent (Lee & Jung, 2015). Workplace stress can also result from nurses’ workload, raising their blood pressure and wearing them out physically and mentally (Zakiyah et al., 2022). This chronic stress significantly contributes to mental health challenges or worsens existing conditions. According to a study by Kang et al. (2020) 17.5% of participants sought out psychological counseling services. In comparison, 36.9% of Wuhan's nursing staff had mental health issues below the threshold, 34.4% had mild mental health issues, and 22.4% had moderate mental health issues (Kang et al., 2020).

Research on Canadian healthcare workers found that among healthcare workers, nurses experienced the highest rate of burnout at 89.5% during the COVID-19 pandemic (Liu et al., 2024). Contributing factors included working directly with COVID-19 patients, fewer years of work experience, lack of workplace support, inadequate leadership, and poor work environment (Liu et al., 2024). Additionally, another study reported demanding workload, lack of staffing that creates poor nurse-to-patient ratios, longer working hours, lack of communication between doctors and nurses, and lack of leadership within nursing structure can also contribute to burnout among nurses (Shah et al., 2021).

The COVID-19 pandemic has significantly increased stress and burnout among nurses, leading many to leave or consider leaving the profession (Canadian Federation of Nurses Union, 2022; Crowe et al., 2022; Registered of Practical Nurses Association of Ontario, 2024). An Ontario study found that 59% of nurses reported an intent to leave during the pandemic (Registered of Practical Nurses Association of Ontario, 2024), while another study revealed that 22% of Canadian critical care nurses considered leaving their profession during this period (Crowe et al., 2022). A survey conducted by the Canadian Federation of Nurses Unions (2022) found that more than half of all nurses were contemplating leaving their position within the next year, with burnout (57%) cited as the primary reason. Other key factors included the inability to provide adequate care (45%), insufficient staffing levels (43%), lack of management support (36%), unpredictable staffing (21%), and lack of recognition (21%) (Canadian Federation of Nurses Union, 2022).

The departure of numerous nurses from the workforce has led to a severe nursing shortage during the pandemic (Lopez et al., 2022; Xu et al., 2020). Though the nursing shortage was apparent pre-COVID (Buerhaus et al., 2007; Marć et al., 2019; Snavely, 2016), the pandemic further exacerbated the problem by introducing additional stressors for nurses (Baumann & Crea-Arsenio, 2023). A nursing shortage significantly impacts the delivery of care, making it more difficult when experiencing a healthcare crisis like the COVID-19 pandemic. High nursing shortages contribute to disrupted continuity of care (Hayes et al., 2006), medical errors (Buchan, 2006; O’Brien-Pallas et al., 2010), patient dissatisfaction (De Simone et al., 2018), and elevated mortality rates (Aiken et al., 2017; Rafferty et al., 2007). A shortfall of nurses is also associated with reduced productivity due to insufficient staffing levels needed to complete tasks efficiently (Jones et al., 2015).

Examining factors associated with intent to leave is imperative in alleviating the nursing turnover rate amidst a public health crisis. While studies have investigated intent to leave among nurses during the pandemic, there is limited research emphasizing intent to leave due to job stress and burnout. Henceforth, this study aims to investigate the demographic and occupational factors associated with intent to leave among Canadian nurses due to burnout and stress during the COVID-19 pandemic. Findings from this study will aid healthcare employers and policymakers in developing interventions to mitigate these risks. These interventions can allow nurses to better prepare for future public health crises, improving their well-being and optimizing patient outcomes.

## Methods and procedures

### Study design

This study employed a quantitative, cross-sectional design using secondary data from the Survey on Health Care Workers’ Experiences During the Pandemic (SHCWEP) conducted by Statistics Canada (2022). The manuscript was developed following the Strengthening the Reporting of Observational Studies in Epidemiology (STROBE) checklist for cross-sectional studies to ensure transparency and methodological rigor.

### Recruitment and data collection

The SHCWEP survey assessed the impact of COVID-19 on Canadian healthcare workers (Statistics Canada, 2022). It collected data on job type and setting, demographic characteristics, personal protective equipment (PPE) and infection prevention and control (IPC) protocols and practices, COVID-19 diagnosis and vaccination status, and the overall impact on life and work due to the pandemic. The survey included individuals aged 18 years or older, randomly selected from the 2016 Census respondents who were classified as healthcare workers based on the National Occupational Classification (NOC) 2016 or were registered in the healthcare education program according to the Classification of Instructional Programs (CIP) 2016. Data collection occurred from September to November 2021 through an online self-administered electronic questionnaire (EQ) or a computer-assisted telephone interview (CATI). However, for the purposes of this study, the analysis was filtered to focus exclusively on data collected from nurses, including nursing coordinators and supervisors, registered nurses, registered psychiatric nurses, and licensed practical nurses. Statistics Canada followed up on respondents who did not complete the survey within a certain period via telephone, email, or text. Out of the 22,292 eligible individuals for the SHCWEP, 12,246 responded, yielding a response rate of 54.9% (Statistics Canada, 2022).

### Dependent variable measures

The study's outcome is intent to leave due to job stress and burnout. Information concerning this outcome is presented as a dichotomous variable in which responses are either “Yes” or “No” indicating whether participants intend to leave their job due to burnout or stress. Two questions were asked concerning job intention in the survey. First, participants were asked, “How long are you planning to stay in your current job?” The participants were given the options of answering either “Less than 6 months”, “6 months to less than a year”, “1 to 2 years”, “3 to 5 years”, “6 or more years”, or “Don’t know”. For those who intended on remaining at their current job for less than 5 years, they were asked a follow up question of “What are the reasons that you might consider leaving or changing your job?” Participants were allowed to choose multiple answers which include, “Retiring”, “Job Stress or Burnout”, “Lack of Job Satisfaction”, “Concerns about your physical health and safety”, “Concerns about your mental health and well-being”, “Concerns about the physical and mental health of household members or others close to you”, “Financial impacts or concerns” or “Long-term impacts of COVID-19 on health care system, including changes in method of delivery of health care”, “Other career opportunity”, “Other”, or “Don’t Know”. Nurses who selected that they would stay at their job for less than five years and indicated that it was due to job stress and burnout were categorized as “Yes”. Job intentions unrelated to stress and burnout were categorized as “No.”

### Independent variable measures

The study examined various demographic and job-related variables to assess their association with intent to leave due to burnout and job stress among nurses. Job-related predictors included age, gender, immigration status, household income, province, visible minority status, and number of household members. Meanwhile, job-related variables consisted of number of locations worked, working experience, job setting, healthcare delivery, training on Infection Prevention and Control (IPC), training on Protective Protection Equipment (PPE), availability of professional emotional support, increase in work hours due to COVID-19, change in workload due to COVID-19, and change in method of delivery due to COVID-19.

### Statistical analysis

Summary statistics were prepared for all explanatory variables, and logistic regression models were generated. The first model examined the association between demographic factors and the intent to leave among nurses. In this model, the intent to leave was the dependent variable, and demographic factors were the independent variables. Similarly, the second model examined the association between job-related factors and the intent to leave among nurses, with job-related factors as the independent variables and intent to leave as the dependent variable. The associations were assessed using odds ratios and their 95% confidence intervals. Missing values were excluded from specific calculation. All analyses were performed using R version 4.4.1 (R Core Team, 2024).

### Ethics

No ethical approval was required at our institution for this study as the data used is publicly available.

## Results

### Descriptive statistics

Out of the 22,292 eligible individuals for the SHCWEP, 12,246 responded, yielding a response rate of 54.9% (Statistics Canada, 2022). A total of 1,138 participants were considered for the analysis after being restricted to nurses and those who answered the intention to leave question. The participants’ sociodemographic characteristics are displayed in Table 1. Most participants (42.35%) reported their age as 55 years or older. The sample also included nurses who mainly identified as female (91.92%). Additionally, most of the nurses in this study (84.53%) were born in Canada. The participants predominantly belonged to low-income households (31.63%), and approximately thirty-two percent of the nurses in this study live in the Atlantic provinces. Most of the participants (85.33%) were not visible minorities. Around forty-one percent of the nurses in the study came from a two-member household.

**Table 1.** Descriptive statistics of demographic predictors of study population.

Variables	Categories	n (n = 1138)	(%)
Age group	18 to 34 years	337 (29.61%)	29.61%
	35 to 44 years	199 (17.49%)	17.49%
	45 to 54 years	118 (10.37%)	10.37%
	55 years and older	482 (42.35%)	42.35%
	N/A*	2 (0.18%)	0.18%
Gender	Female	1046 (91.92%)	91.92%
	Male	90 (7.91%)	7.91%
	N/A*	2 (0.18%)	0.18%
Immigration status	Canadian-born	962 (84.53%)	84.53%
	Immigrant	124 (10.90%)	10.90%
	N/A*	52 (4.57%)	4.57%
Household income	$150,000+	305 (26.80%)	26.80%
	$100,000–$149,999	280 (24.60%)	24.60%
	Under $100,000	360 (31.63%)	31.63%
	N/A	193 (16.96%)	16.96%
Province	Alberta	128 (11.25%)	11.25%
	Atlantic	361 (31.72%)	31.72%
	British Columbia	164 (14.41%)	14.41%
	Manitoba	130 (11.42%)	11.42%
	Ontario	129 (11.34%)	11.34%
	Saskatchewan	114 (10.02%)	10.02%
	Quebec	112 (9.84%)	9.84%
Visible minority status	Yes	131 (11.51%)	11.51%
	No	971 (85.33%)	85.33%
	N/A	36 (3.16%)	3.16%
Number of household members	One	150 (13.18%)	13.18%
	Two	471 (41.39%)	41.39%
	Three	176 (15.47%)	15.47%
	Four and more	259 (22.76%)	22.76%
	N/A*	82 (7.21%)	7.21%

*N/A = Not Applicable.

Table 2 shows the job-related characteristics of the participants. Most of the nurses in the study (66.96%) reported working at one location. Concerning work experience, approximately thirty-nine percent of the participants had less than ten years of experience. Over half of the nurses in the study (58.79%) worked in an acute care setting. Most participants received training on IPC and PPE (87.79% and 83.74%, respectively). Approximately fifty-one percent of the nurses in the study stated that they “disagreed” that professional emotional support was available at their workplace for those who required it. Most participants (56.85%) experienced increased work hours due to the COVID-19 pandemic. Additionally, a significant number of nurses (76.45%) reported changes in workload in response to the COVID-19 pandemic. About seventy-five percent of the nurses did not experience changes in the method of delivery due to the COVID-19 pandemic. This meant that there were no modifications in how nursing care or services were delivered, such as shifts from in-person to virtual consultations or changes in procedures.

**Table 2.** Descriptive statistics for job-related predictors.

Variables	Categories	n (n = 1138)	(%)
Number of locations worked	One	762	66.96%
	Multiple	328	28.82%
	N/A*	48	4.22%
Number of working experience years of work experience	20 or more	410	36.03%
	10 to 19	199	17.49%
	Less than 10	440	38.66%
	N/A*	89	7.82%
Type of job setting	Outpatient and ambulatory care	168	14.76%
	Acute care	669	58.79%
	Community/Homecare	58	5.10%
	Long-term care	164	14.41%
	N/A*	79	6.94%
Type of healthcare delivery	Virtual	37	3.25%
	Hybrid	116	10.19%
	In-Person	912	80.14%
	N/A*	73	6.41%
Received formal training on IPC†	Yes	999	87.79%
	No	96	8.44%
	N/A*	43	3.79%
Received formal training on PPE¶	Yes	953	83.74%
	No	103	9.05%
	N/A*	82	7.21%
Professional emotional support was available to those who needed it	Agree	411	36.12%
	Disagree	363	31.90%
	N/A* or neutral	364	31.99%
Increase in work hours due to COVID-19 pandemic	Yes	647	56.85%
	No	489	42.97%
	N/A*	2	0.18%
Change in workload due to COVID-19 pandemic	Yes	870	76.45%
	No	266	23.37%
	N/A*	2	0.18%
Change in method of delivery due to COVID-19 pandemic	Yes	279	24.51%
	No	857	75.31%
	N/A*	2	0.18%

*N/A = Not Available (Missing Values); †IPC = Infection Prevention and Control; ¶PPE = Personal Protective Equipment.

### Sociodemographic factors associated with intent to leave

Multivariate logistic regression was used to generate odds ratios to examine the demographic predictors associated with intent to leave among nurses (Table 3). Based on the results, age, and province were statistically significant predictors. Nurses who belonged to the age group 18–34 and 35–44 were 9.39 times and 9.95 times more likely to consider leaving their profession due to job stress and burnout compared to those who were in the 55+ age group [OR = 9.39, 95% CI: (6.25–14.12); OR = 9.95, 95% CI: (5.92–16.73)]. Moreover, nurses living in Alberta and British Columbia are both 3.16 times more likely to consider leaving their jobs due to job stress and burnout to those living in Quebec [OR = 3.16, 95% CI: (1.58–6.32); OR = 3.16, 95% CI: (1.66–6.03)].

**Table 3.** Demographic factors associated with intent to leave among nurses amidst the COVID-19 pandemic (n = 1138).

Variables	Categories	Odds Ratio	95% CI
Age	55+	1.00 (Ref)	N/A
	45–54	4.94**	2.94–8.28
	35–44	9.95**	5.92–16.73
	18–34	9.39**	6.25–14.12
Gender	Female	1.00 (Ref)	N/A
	Male	1.01	0.312–3.31
Province	Quebec	1.00 (Ref)	N/A
	Alberta	3.16**	1.58–6.32
	Atlantic provinces	1.63**	0.93–2.84
	British columbia	3.16**	1.66–6.03
	Manitoba	2.84**	1.39–5.82
	Ontario	2.63**	1.36–5.07
	Saskatchewan	2.16**	1.06–4.37
Income	$500,000+	1.00 (Ref)	N/A
	$100,000–$149,999	1.24	0.82–1.87
	Under $100,000	1.25	0.82–1.89
Immigration status	Immigrant	1.00	N/A
	Canadian-borne	1.16	0.52–2.56
Household members	Three	1.00 (Ref)	N/A
	Four	1.46	0.88–2.42
	Two	1.36	0.87–2.15
	One	1.49	0.77–2.52
Visible minority status	No	1.00 (Ref)	N/A
	Yes	1.09	0.49–2.43

**p-value < .05.

A second logistic regression analysis was conducted to obtain odds ratios for job-related factors associated with intent to leave among nurses (Table 4). Work experience, type of job setting, availability of professional emotional support, change in workload, and increase in work hours were significantly associated with intent to leave among nurses. Nurses with work experience of 10 to 19 years and less than ten years were 3.90 and 3.91 times more likely to consider leaving their profession compared to those with work experience of over 20 years (OR 3.90, 95 CI 2.33–6.53; OR 3.91, 95 CI 2.53–6.05). Moreover, nurses working in an acute care setting had 3.31 times greater odds of intending to leave the nursing profession than those who work in outpatient and ambulatory care (OR 3.31, 95 CI 1.69–6.51). Participants who believed there was a lack of professional emotional support at their workplace had 3.43 times higher odds of intending to leave their profession than those satisfied with the amount of emotional support (OR 3.43, 95 CI 2.31–5.09). Nurses who experienced changes in workload due to the pandemic were 2.69 times more likely to consider leaving their jobs due to burnout and job stress than those who did not experience any changes [OR = 2.69, 95% CI: (1.58–4.57)]. Additionally, nurses who reported increased work hours due to the COVID-19 pandemic were 1.92 times more likely to intend to leave their jobs because of burnout and job stress [OR = 1.92, 95% CI: (1.27–2.92)].

**Table 4.** Job-related factors associated with intent to leave among nurses amidst the COVID-19 pandemic (n = 1138).

Variables	Categories	Odds Ratio	95% CI
Number of locations worked	Multiple	1.00 (Ref)	N/A
	One	1.08	0.69–1.69
Years of work experience	20+	1.00 (Ref)	N/A
	10–19	3.90**	2.33–6.53
	Less than 10	3.91**	2.53–6.05
Type of job setting	Outpatient and ambulatory care	1.00 (Ref)	N/A
	Acute care	3.31**	1.69–6.51
	Community/Homecare	2.33	0.80–6.79
	Long-term care	1.98*	0.89–4.42
Delivery of healthcare services	Virtual	1.00 (Ref)	N/A
	Hybrid	1.79	0.35–9.09
	In-Person	1.27	0.28–5.80
Received formal training on IPC†	Yes	1.00 (Ref)	N/A
	No	1.81	0.83–3.94
Received formal training on PPE¶	No	1.00 (Ref)	N/A
	Yes	1.45	0.68–3.06
Professional emotional support was available to those who required it	Agree	1.00 (Ref)	N/A
	Disagree	3.43**	2.31–5.09
Change in workload due to COVID-19 pandemic	No	1.00 (Ref)	N/A
	Yes	2.69**	1.58–4.57
Increase in work Hours due to COVID-19 pandemic	No	1.00 (Ref)	N/A
	Yes	1.92**	1.27–2.92
Change in method of healthcare delivery due to COVID-19 pandemic	No	1.00 (Ref)	N/A
	Yes	1.01	0.60–1.71

*p-value < .1; **p-value < .05; †IPC = Infection Prevention and Control; ¶PPE = Personal Protective Equipment.

## Discussion

The COVID-19 pandemic was a particularly stressful time for nurses as they had to respond rapidly to the public's health needs. Though their efforts were integral in controlling the spread and treating patients, many nurses reported experiencing immense burnout and job stress, leading several to consider leaving their jobs. The study aimed to identify demographic and occupational predictors of Canadian nurses’ intent to leave due to burnout and job stress during the COVID-19 pandemic. The findings revealed that age and province were significant demographic predictors of nurses’ intent to leave. Additionally, work experience, job setting, availability of professional emotional support, changes in workload due to the pandemic, and increased work hours were significant occupational predictors.

We found that younger nurses were at a higher risk of intending to leave their profession due to burnout and job stress compared to older nurses, aligning with previous research (Koehler & Olds, 2022; Raso et al., 2021; Sihvola et al., 2023; Tadesse et al., 2023). For instance, a cross-sectional study involving 437 registered nurses from Finland found that those among the oldest age group (51–69 years) had significantly higher overall job satisfaction and less intention to leave during the pandemic than younger nurses (Sihvola et al., 2023). Younger nurses are more likely to experience burnout during the COVID-19 pandemic due to their inexperience in handling extreme situations, leading them to consider leaving the profession as they feel unprepared (Tam et al., 2004). In contrast, older nurses with greater experience can better adapt to rapid changes and may not feel the same need to leave the profession despite the immense stress. Furthermore, generational differences may also contribute to this trend, as younger nurses typically prioritize work-life balance more than older generations (Tan & Chin, 2023).

Intent to leave among nurses is consistent across provinces in Canada, reflecting findings from previous studies. A study of 1,705 frontline nurses in Quebec found that those caring for COVID-19 patients reported high intentions to leave due to inadequate preparation and feeling overwhelmed at work (Lavoie-Tremblay et al., 2022). Another Quebec-based study found that nearly 50% of nurses intended to leave the profession before reaching the age of 35 (Faubert, 2023). Similarly, research by D’Alessandro-Lowe et al. (2024), which primarily surveyed Ontario nurses (62.3%), reported that high workloads and insufficient workplace support negatively impacted nurses’ mental health, ultimately contributing to their intent to leave.

Our results also showed that nurses with less experience in the field are at higher odds of intending to leave compared to those with a greater amount of work experience. Raso et al. (2021) reported similar findings, showing that (i.e., 25+ years) had less intention to leave the industry during the pandemic compared to those with the least experience (i.e., less than two years). Contrastingly, those under 29 experienced less of an intent to leave compared to their 60+ year old counterparts (Raso et al., 2021). Similar to what was found for age, nurses with greater experience are typically able to handle stressful situations more effectively, as they are accustomed to the profession's demands (Tam et al., 2004). Conversely, those with less experience may find it more challenging to adapt.

While many nurses experienced burnout during the pandemic, certain sectors of nurses were more deeply impacted than others, specifically those who worked in acute care or intensive care units (ICU). ICU nurses have historically faced high prevalence rates of burnout compared to other units due to the demanding and high-stress nature of their work (Fischer et al., 2020). Research has shown that burnout and intention to leave the profession increased in ICU nurses amidst the pandemic (Bruyneel et al., 2021; Butera et al., 2021; Vermeir et al., 2018). Within Ontario, from March 20, 2020, to October 31, 2021, nearly 10,000 Ontarians had been admitted to the ICU with COVID-19-related illness, and within the peak of the pandemic's third wave, the number of patients on ventilators was 180% higher than pre-pandemic averages (Barrett et al., 2021). This high influx of patients required ICU nurses to make vast adjustments to manage the increased workload while also ensuring their own safety amid infection risk. The prolonged fatigue (Firew et al., 2020; Shen et al., 2020) from these increased demands and the constant risk of infection continue to impact many ICU nurses to intend leaving the healthcare sectore (Petrișor et al., 2021).

Nurses who lacked access to professional mental support had higher odds of reporting intentions to leave nursing compared to those who felt their workplace provided sufficient support. Nurses who lacked professional mental support availability were more at odds of reporting intentions to leave nursing than those who felt that their workplace had sufficient mental support. Studies have reported that psychological support can aid healthcare workers in managing their feelings and emotions more effectively during a claustrophobic event (Balicer et al., 2006; Mymin Kahn et al., 2016). These findings underscore the importance of having professional emotional support available for workers in the workplace. Likewise, studies have also shown the significance of social support availability for healthcare workers (Chan, 2004; Galanis et al., 2021; Naushad et al., 2019). A review conducted by Galanis et al. (2021) indicated that decreased social support was linked to increased burnout among nurses during the COVID-19 pandemic. This was because social support allows nurses to control and avoid any negative feelings, decreasing the likelihood of burnout syndrome (Galanis et al., 2021). Other studies have highlighted that strong social support networks during the pandemic can alleviate feelings of isolation and improve turnover intention among healthcare workers (Hou et al., 2020; Southwick & Southwick, 2020; Wu et al., 2020).

The findings revealed that nurses who reported changes in their workload during the pandemic were more likely to intend to leave than those who did not report any changes. The lack of hospital staffing to deliver high-quality, evidence-based nursing care is a persistent criticism among nurses worldwide (Aiken et al., 2017; Griffiths et al., 2018). Unreasonable workloads are imposed on nurses in hospitals due to the growing shortage of nursing staff. Nurses frequently handle seven to eight patients with severe illnesses who are at significant risk of fatalities or complications due to unreasonable workloads (Winsett et al., 2016). A recent survey on job satisfaction found that 55% of nurses said they did not have sufficient time to devote to their patients (Phillips, 2020). Workflow disruptions, severity of patients, nurse-patient proportions, a lack of organizational assistance, and inadequate staffing numbers have all been identified as variables affecting workload (Tarcan et al., 2017). In another study examining the working circumstances of 33, 659 nurses from 488 European hospitals, researchers discovered that basic nursing responsibilities were neglected because of an increasing workload (Phillips, 2020). In this group of nurses, increasing workloads combined with low job satisfaction led to higher rates of burnout and turnover in employment (Khan et al., 2019; Sobaski, 2018; Winsett et al., 2016). Nurses experienced dissatisfaction, disengagement, and emotional exhaustion when they do not receive sufficient assistance to manage workload (Bureau of Labor Statistics, 2024).

Nurses who were subjected to increased work hours during the pandemic had higher odds of intent to leave. Long work hours and shift work are common in nursing (Dall’Ora et al., 2023). There are no national work-hour policies for registered nurses, despite restrictions on the duration of shifts and total working hours for residents in medical programs and other professions (Stimpfel et al., 2012). Furthermore, the exact duration of shifts is frequently uncertain due to variations in patient requirements and unforeseen personnel adjustments (Rogers et al., 2004). A study by Bae (2024), involving 264 nursing staff across 28 hospitals, discovered 40% claimed to have worked required overtime in the last month. Additionally, among those required to work overtime, 40–60% of respondents said they routinely missed meals and breaks while caring for patients and felt confined to complete their duties (Chang et al., 2005). These issues contributed to greater discontent with their workplace and a lack of ability to care for themselves. Dissatisfaction with care for patients increased as the percentage of hospital nurses employed for shifts longer than thirteen hours increased (Stimpfel et al., 2012). Additionally, nurses who worked ten hours or longer shifts had a 25% higher chance of burnout, job dissatisfaction, and intention to quit than nurses who worked shorter shifts (Stimpfel et al., 2012), whereby demand for overtime resulted in a rise in sickness, injuries, and absenteeism (Bae & Fabry, 2014; Hayes et al., 2006).

This study presented several strengths. Firstly, the large sample size of 1,138 participants decreases the margin of error, leading to more precise estimates of the odds ratio. Second, to our knowledge, this is the first study to examine factors associated with intent to leave among nurses due to job stress and burnout within a Canadian COVID-19 context. Findings from this study will be critical in devising strategies to mitigate these risks amidst a future public health crisis.

Despite these strengths, some limitations should be acknowledged. One limitation is the lack of diversity considerations. Our study sample primarily consisted of nurses not belonging to a visible minority group (85.33%). With the lack of representation from minority groups, the study failed to capture their experiences during the pandemic and their impact, thereby hindering the development of tailored interventions for this population. Another limitation concerns the assessment of burnout. Burnout is not merely defined as severe stress, rather it is characterized by three dimensions: exhaustion, cynicism, and decreased professional efficacy (Maslach et al., 2001). Due to its complexity, tools such as the Maslach Burnout Inventory (MBI) are used to measure and assess burnout across these dimensions accurately (Maslach et al., 1997). However, this study assessed burnout through a yes/no question that fails to capture the intricate nuances and multifaceted nature of burnout. Moreover, the reliance on self-reported data introduces the potential for information bias, as responses may be affected by individual perceptions, memory recall, and social desirability. Additionally, the sample does not accurately reflect Canada's population, as it overrepresents participants from less populated regions, such as the Atlantic provinces while underrepresenting those from highly populated areas like Ontario and Quebec, with only 11.34% and 9.84% of participants from these provinces, respectively. This limitation, in turn, affects the generalizability of the findings.

## Conclusion

Individual factors, such as age and province, were significantly associated with Canadian nurses’ intent to leave due to job stress and burnout amidst the COVID-19 pandemic. Additionally, occupational factors, such as work experience, job setting, emotional support availability, changes in workload, and increased work hours were significant predictors. These study's findings are crucial for devising strategies (e.g., implementing mental health support programs, establishing peer support networks, offering regular breaks, adjusting patient-to-nurse ratios, providing flexible shift schedules, and ensuring clear communication regarding workload expectations) that can mitigate the risk of nurses leaving the profession during a public health crisis. Nurses played a key role in managing the pandemic by treating patients and educating the public on health and safety. Given their importance, efforts should be made to retain as many nurses as possible, as this can improve the healthcare system and the population's overall health. Future research should focus on assessing intent to leave due to burnout and job stress involving more ethnic minorities and using tools such as MBI to allow for a more accurate understanding of burnout. Also, a mixed-methods approach that includes qualitative data should be considered to explore nurses’ personal experiences in greater depth.

## Supplemental Material

sj-docx-1-cjn-10.1177_08445621251338580 - Supplemental material for Factors Associated with Intent to Leave and Burnout among Canadian Nurses Amidst the COVID-19 Pandemic: A Quantitative Analysis of the Survey on Health Care Workers’ Experiences During the Pandemic Supplemental material, sj-docx-1-cjn-10.1177_08445621251338580 for Factors Associated with Intent to Leave and Burnout among Canadian Nurses Amidst the COVID-19 Pandemic: A Quantitative Analysis of the Survey on Health Care Workers’ Experiences During the Pandemic by Kishana Balakrishnar, Bao-Zhu Stephanie Long, Alexia M. Haritos, Edris Formuli and Behdin Nowrouzi-Kia in Canadian Journal of Nursing Research

sj-docx-2-cjn-10.1177_08445621251338580 - Supplemental material for Factors Associated with Intent to Leave and Burnout among Canadian Nurses Amidst the COVID-19 Pandemic: A Quantitative Analysis of the Survey on Health Care Workers’ Experiences During the Pandemic Supplemental material, sj-docx-2-cjn-10.1177_08445621251338580 for Factors Associated with Intent to Leave and Burnout among Canadian Nurses Amidst the COVID-19 Pandemic: A Quantitative Analysis of the Survey on Health Care Workers’ Experiences During the Pandemic by Kishana Balakrishnar, Bao-Zhu Stephanie Long, Alexia M. Haritos, Edris Formuli and Behdin Nowrouzi-Kia in Canadian Journal of Nursing Research

## Data Availability

The datasets used and/or analyzed during the current study are available upon request from the corresponding author.
