# Supplementary material for: Factors Associated with Intent to Leave and Burnout among Canadian Nurses Amidst the COVID-19 Pandemic: A Quantitative Analysis of the Survey on Health Care Workers’ Experiences During the Pandemic
Source: Can J Nurs Res. 2025 Apr 29;57(3):448–59. doi: 10.1177/08445621251338580 (PMC12344211; doi:10.1177/08445621251338580)
Supplement: sj-docx-1-cjn-10.1177_08445621251338580 - Supplemental material for Factors Associated with Intent to Leave and Burnout among Canadian Nurses Amidst the COVID-19 Pandemic: A Quantitative Analysis of the Survey on Health Care Workers’ Experiences During the Pandemic [file sj-docx-1-cjn-10.1177_08445621251338580.docx]

**Supplementary Material**

**Table 1.** Descriptive Statistics of Demographic Predictors of Study Population and Entire Dataset.

| **Variables** | **Categories** | **Study Population (%)** | **Dataset (%)** |
| --- | --- | --- | --- |
| Age Group | 18 to 34 years | 337 (29.61%) | 2603 (21.44%) |
|  | 35 to 44 years | 199 (17.49%) | 3037 (25.02%) |
|  | 45 to 54 years | 118 (10.37%) | 2906 (23.94%) |
|  | 55 years and older | 482 (42.35%) | 3553 (29.27%) |
|  | N/A | 2 (0.18%) | 40 (0.33%) |
| Gender | Female | 1046 (91.92%) | 9317 (76.75%) |
|  | Male | 90 (7.91%) | 2785 (22.94%) |
|  | N/A | 2 (0.18%) | 37 (0.30%) |
| Immigration Status | Canadian-born | 962 (84.53%) | 9116 (75.10%) |
|  | Immigrant | 124 (10.90%) | 2170 (17.88%) |
|  | N/A | 52 (4.57%) | 853 (7.03%) |
| Household Income | $150,000+ | 305 (26.80%) | 4058 (33.43%) |
|  | $100,000-$149,999 | 280 (24.60%) | 2016 (16.61%) |
|  | Under $100,000 | 360 (31.63%) | 3741 (30.81%) |
|  | N/A | 193 (16.96%) | 2324 (19.14%) |
| Province | Alberta | 128 (11.25%) | 1457 (12.00%) |
|  | Atlantic | 361 (31.72%) | 3529 (29.07%) |
|  | British Columbia | 164 (14.41%) | 1531 (12.61%) |
|  | Manitoba | 130 (11.42%) | 1413 (11.64%) |
|  | Ontario | 129 (11.34%) | 1426 (11.75%) |
|  | Saskatchewan | 114 (10.02%) | 1283 (10.57%) |
|  | Quebec | 112 (9.84%) | 1500 (12.35%) |
| Visible Minority Status | Yes | 131 (11.51%) | 2032 (16.74%) |
|  | No | 971 (85.33%) | 9203 (75.81%) |
|  | N/A | 36 (3.16%) | 904 (7.44%) |
| Number of Household Members | One | 150 (13.18%) | 1170 (9.64%) |
|  | Two | 471 (41.39%) | 3498 (28.82%) |
|  | Three | 176 (15.47%) | 1918 (15.80%) |
|  | Four and more | 259 (22.76%) | 4072 (33.54%) |
|  | N/A | 82 (7.21%) | 1481 (12.20%) |

* N/A = Not Applicable

**Table 2.** Descriptive Statistics for Job-Related Predictors for Study Population and Entire Dataset

| **Variables** | **Categories** | **Study Sample (%)** | **Dataset (%)** |
| --- | --- | --- | --- |
| Number of Locations Worked | One | 762 (66.96%) | 6989 (57.57%) |
|  | Multiple | 328 (28.82%) | 4425 (36.45%) |
|  | N/A | 48 (4.22%) | 725 (5.97%) |
| ~~Number of Working Experience~~ Years of Work Experience | 20 or more | 410 (36.03%) | 3515 (28.96%) |
|  | 10 to 19 | 199 (17.49%) | 3219 (26.52%) |
|  | Less than 10 | 440 (38.66%) | 4000 (32.95%) |
|  | N/A | 89 (7.82%) | 1405 (11.57%) |
| Type of Job Setting | Outpatient and Ambulatory Care | 168 (14.76%) | 2579 (21.25%) |
|  | Acute Care | 669 (58.79%) | 4547 (37.46%) |
|  | Community/Homecare | 58 (5.10%) | 1105 (9.10%) |
|  | Long-term Care | 164 (14.41%) | 2015 (16.60%) |
|  | N/A | 79 (6.94%) | 1893 (15.59%) |
| Type of Healthcare Delivery | Virtual | 37 (3.25%) | 443 (3.64%) |
|  | Hybrid | 116 (10.19%) | 2626 (21.63%) |
|  | In-Person | 912 (80.14%) | 8100 (66.73%) |
|  | N/A | 73 (6.41%) | 970 (7.99%) |
| Received Formal Training on IPC† | Yes | 999 (87.79%) | 9929 (81.79%) |
|  | No | 96 (8.44%) | 1636 (13. 48%) |
|  | N/A | 43 (3.79%) | 574 (4.73%) |
| Received Formal Training on PPE¶ | Yes | 953 (83.74%) | 9362 (77.12%) |
|  | No | 103 (9.05%) | 1706 (14.05%) |
|  | N/A | 82 (7.21%) | 1071 (8.82%) |
| Professional Emotional Support was Available to those who needed it | Agree | 411 (36.12%) | 5433 (44.76%) |
|  | Disagree | 579 (50.88%) | 2711 (22.33%) |
|  | N/A or neutral | 364 (31.99%) | 3995 (32.91%) |
| Increase in Work Hours due to COVID-19 Pandemic | Yes | 647 (56.85%) | 5665 (46.67%) |
|  | No | 489 (42.97%) | 6367 (52.45%) |
|  | N/A | 2 (0.18%) | 107 (0.88%) |
| Change in workload due to COVID-19 Pandemic | Yes | 870 (76.45%) | 8133 (67.00%) |
|  | No | 266 (23.37%) | 3899 (32.12%) |
|  | N/A | 2 (0.18%) | 107 (0.88%) |
| Change in method of Delivery due to COVID-19 Pandemic | Yes | 279 (24.51%) | 3929 (32.37%) |
|  | No | 857 (75.31%) | 8103 (66.75%) |
|  | N/A | 2 (0.18%) | 107 (0.88%) |

* N/A = Not Available (Missing Values); † IPC = Infection Prevention and Control; ¶ PPE = Personal Protective Equipment
